# Therapeutic drug monitoring for antimicrobial agents for people living with HIV (TAP)

**DOI:** 10.12688/wellcomeopenres.22707.2

**Published:** 2025-04-22

**Authors:** Christine Sekaggya-Wiltshire, Eva Agnes Laker Odongpiny, Francis Williams Ojara, Isabella Kyohairwe, Reuben Kiggundu, Hope Mackline, Catriona Waitt, Aida N Kawuma, Allan Kengo, Allan Buzibye, Noela Owarwo, Francis Kakooza, Andrew Kambugu

**Affiliations:** 1 College of Health Sciences, Makerere University Infectious Diseases Institute, Kampala, Central Region, Uganda; 2 University of Liverpool School of Medicine, Liverpool, England, UK

**Keywords:** Therapeutic Drug Monitoring, Pharmacokinetics, Pharmacodynamics, dose adjustment, Antimicrobial Agents, HIV, Tuberculosis

## Abstract

**Background:**

Antimicrobial resistance (AMR) is a growing health concern, particularly in Africa, and is predicted to become the leading cause of death after cancer by 2050. Factors like overuse or inappropriate use of antibiotics contribute to this crisis. People living with HIV (PLWH) are particularly vulnerable to AMR with potential drug-drug interactions between antiretroviral and antimicrobial agents against common organisms like *Mycobacterium tuberculosis.* There is limited data on the concentrations of commonly used antimicrobial agents in people living with HIV in resource-limited settings. Therapeutic Drug Monitoring (TDM) offers a promising approach to optimize antibiotic dosing and improve treatment outcomes for those with sub-optimal drug concentrations. TDM has been recommended for PLWH on anti-tuberculosis treatment due to sub-optimal drug concentrations found in a significant proportion of those with TB.

**Objectives:**

The main objectives of this study are to determine the concentrations of selected antimicrobial agents in people living with HIV requiring antimicrobial therapy and to assess the utility of therapeutic drug monitoring in achieving therapeutic targets for PLWH receiving rifampicin and isoniazid for the treatment of tuberculosis.

**Methods:**

This prospective observational study will enroll adult PLWH receiving amoxicillin, azithromycin, ciprofloxacin, rifampicin, isoniazid, or ceftriaxone. Concentrations of these antibiotics will be measured locally using validated liquid chromatography mass spectrometry methods and high-performance liquid chromatography with ultraviolet detection. TDM with dose adjustment will be performed in a subset of participants on TB treatment. Pharmacokinetic parameters will be estimated using non-linear mixed effects models.

**Results:**

This study was reviewed and approved by the research and ethics committee in February 2024. Participant enrolment began in September 2024.

**Conclusions:**

We anticipate that the findings from this research will characterize pharmacokinetic and pharmacodynamics relationships to predict treatment response for optimal antimicrobial therapeutic and anti-tuberculosis dosing among people living with HIV (PLWH).

**Clinical registration:**

The study is registered with Pan African Clinical Trials Registry, registration number PACTR202409710100607, registration date 07 August 2024, pactr.samrc.ac.za/TrialDisplay.aspx?TrialID=31764

## Plain Language Summary

Antimicrobial resistance is on the increase and people living with HIV may be at increased risk for this, including those living with HIV due to interactions between antiretroviral and antibiotics. There is scarce data about the concentrations of the commonly used antibiotics in the blood of people living with HIV. Monitoring the drug concentrations and adjusting the dose when needed has been recommended in people living with HIV who develop TB. The main objectives of this study are to determine the concentrations of selected antibiotics in people living with HIV and to assess how useful it is to monitor the amount of the antibiotics in blood while adjusting the dose. This study will enroll adults living with HIV receiving amoxicillin, azithromycin, ciprofloxacin, rifampicin, isoniazid, or ceftriaxone. Concentrations of these antibiotics will be measured, and for those on TB drugs, doses will be adjusted until the participant achieves suitable amounts of the drug in blood. This study was reviewed and approved by the research and ethics committee and we anticipate that the findings from this research will help us to know how much of the antibiotics is in the body and if people respond as expected to the treatment.

## Introduction

Antimicrobial resistance (AMR) is on the rise and is predicted to become the leading cause of after cancer by 2050, with Africa bearing a significant proportion of this burden ^1^. In 2019, an estimated 1.27 million deaths were attributed to antimicrobial-resistant infections, and most of these infections were bacterial ^1^.

Optimal dosing of antimicrobial agents is essential to achieve therapeutic concentration, maximize efficacy, and minimize both resistance and toxicity. Conversely, suboptimal drug levels may cause treatment failure, AMR, and mortality, while excessive exposures may increase toxicity and affect adherence ^2^. In sub-Saharan Africa, overlapping diseases lead to multi-morbidity with altered physiologic states, like those seen in infections or inflammation, and obesity ^3^. These require co-treatment with multiple regimens which may result in drug-drug interactions (DDI) that are often inadequately characterized. The SOUTH study found sub-therapeutic levels of rifampicin and isoniazid amongst TB-HIV coinfected patients, which were associated with delayed sputum conversion ^4^ and, as seen in other studies, TB relapse ^5^. Such findings have prompted trials of higher anti-TB doses to improve outcomes and prevent resistance ^6– 8^. Similarly, suboptimal antibiotic exposure has been associated with poor treatment outcomes ^9^ and development of resistance ^10^.

People living with HIV (PLWH) are at an increased risk of AMR across a range of pathogens and drug classes ^11^. Antibiotics are frequently prescribed in PLWH to manage co-morbidities like other sexually transmitted- and urinary tract infections as well as respiratory tract infection. Prescription rates of these vary widely, ranging from about 40% in Nigeria to as high as 85% on Tanzania ^12^. While this data was not available for Uganda, a recent general survey showed widespread antibiotic use across hospitals ^13^. Outcome data for common infections indicate an 80% treatment success rate for pneumonia, a major cause of mortality amongst especially children in Uganda. However, resistance to commonly used antibiotics is on the rise in Uganda, with reported rates exceeding 70% for ceftriaxone and ampicillin, and about 43% for ciprofloxacin ^14^. While it remains unclear to what extent suboptimal drug exposures directly contribute to these resistance patterns, previous studies have reported inadequate antibiotic concentrations to drive development of AMR ^10^. Other drivers of AMR include genetic mechanisms which also play a significant role in the emergence and spread of resistance to antimicrobials ^15^.

Despite the potential contribution of inadequate drug exposure to AMR, the pharmacokinetic (PK) profiles of commonly prescribed antibiotics in PLWH are not well characterized. Ceftriaxone and amoxicillin may not exhibit significant DDI with ART but some ARVs like ritonavir-boosted protease inhibitors can inhibit transporters like p-glycoprotein and multi-drug resistance protein 2, potentially increasing azithromycin exposure and raising the risk of arrythmias ^16^. However, no clinical PK studies have been conducted to investigate these potential interactions, and dose adjustments are not currently recommended.

Therapeutic drug monitoring (TDM) uses plasma drug concentrations to assess whether a given dose achieves optimal drug exposure ^17^. It helps evaluate attainment of PK targets for the antibiotics and provides objective data to guide dosing decisions. These PK targets are often based on drug exposure parameters like the area under the concentration-time curve (AUC) or peak/trough concentrations relative to the minimum inhibitory concentration (MIC) of the pathogen. For example, azithromycin and ciprofloxacin require AUC/MIC ratios of ≥5 ^18^ and ≥125 ^19^, respectively, while ceftriaxone has a minimum threshold of 8 mg/L ^20^. Amoxicillin efficacy depends on maintaining concentrations above the MIC for at least 40% of the dosing interval ^21^. However, MIC testing is rarely done in resource-limited settings due to cost constraints. For important ant-TB drugs like isoniazid and rifampicin, peak plasma concentration of 3 to 6 mg/L and > 8 mg/L are desired, respectively ^17^.

In resource-limited settings, where antibiotic options are often scarce, optimizing existing treatments is essential to maintain accessibility and prevent AMR. While strategies like regimen intensification, individualized drug combinations, and precision dosing can help prevent AMR, there is limited understanding of which model will be most effective for widespread implementation in low- and middle-income countries (LMICs). TDM has been successfully used for aminoglycosides and anti-TB drugs in special populations, such as PLWH and those with diabetes ^17^. However, there is limited data on its role in optimizing antibiotic therapy for PLWH in LMICs.

## Protocol

### Study objectives

1. To determine the concentrations of selected antimicrobial agents in people living with HIV requiring antimicrobial therapy.
2. To assess the utility of therapeutic drug monitoring in achieving therapeutic targets for people living with HIV receiving rifampicin and isoniazid for treatment of tuberculosis.
3. To develop and validate a population pharmacokinetic-pharmacodynamic model for use during therapeutic drug monitoring for selected antibiotics used among PLWH

### Study outcomes

***On antibiotics***

**Primary endpoints**

Pharmacokinetic parameters

- Trough concentrations (Ctrough) for amoxicillin, ciprofloxacin, azithromycin and ceftriaxone

Pharmacodynamic parameters

Clinical response to antibiotics through the time of treatment will be assessed. Clinical response is either clinical cure (resolution of symptoms) or clinical failure (lack of improvement in signs and symptoms of infection OR recurrence of signs/symptoms of infection after initial improvement.

***On TB treatment***

Pharmacokinetic parameters

- Maximum concentrations (Cmax) and area under the concentration-time curve (AUC) defined as the total exposure for rifampicin, isoniazid

Pharmacodynamic parameters

1. Clinical response will be assessed according to the World Health Organization definitions of cure, completed and treatment failure, which will be extracted from the clinic database.
2. Occurrence of adverse drug reactions

## Methods

### Study site

The study will be conducted at the Infectious Diseases Institute (IDI) which is an out-patient clinic in Kampala, Uganda, where around 8000 PLWH are currently being provided with care, of whom 250 are initiated on TB treatment annually. Participants who are on intravenous medication will be enrolled at Mulago National Referral Hospital which handles both medical and surgical in-patients.

### Study participants

Participants will be included if they fulfil the following criteria:

***Inclusion criteria***

1. A personally signed and dated informed consent document
2. An adult of 18 years and above living with HIV
3. Patients being initiated on or currently receiving antibiotics which may include: amoxicillin (+/- clavulanic acid), azithromycin, ciprofloxacin, rifampicin, isoniazid, ceftriaxone
4. Participants who are willing and able to comply with scheduled visits, treatment plan, laboratory tests, and other study procedures.

***Exclusion criteria***

1. Patients who discontinue antibiotics for any reason following initiation
2. Patients receiving antibiotics only for prophylaxis

### Sample size

We conducted a priori stochastic simulation and estimation to determine the appropriate sample size for our study. Assuming a 30% coefficient of variation (%CV) in drug exposure, we estimated that enrolling 40 participants per antibiotic would provide sufficient power to detect a 30% effect of HIV co-treatment on antibiotic exposure. In alignment with the ICH E9 guidelines on statistical principles for clinical trials and considering an anticipated 10–15% attrition rate due to study withdrawal and loss to follow-up, we increased the sample size by 10 participants per antibiotic. This adjustment results in a total of 50 participants per antibiotic, or 200 participants overall.

For intravenous antibiotics (ceftriaxone) and anti-TB drugs (rifampicin and isoniazid), we assumed a lower %CV of 25% in drug exposure. Based on this assumption and accounting for an anticipated 15% withdrawal rate, we estimate that a sample size of 25 participants will be sufficient to maintain the power needed to detect possible significant changes in drug exposure due to concomitant antiretroviral therapy.

Therefore, the total sample size for this study is 200 participants.

## Study design

### Participants on antimicrobial drugs

This will be a prospective observational study that will include adult patients with suspected or confirmed bacterial infection receiving amoxicillin (+/- clavulanic acid), azithromycin, ciprofloxacin. The decision to start antibiotics will be left to the managing clinician (based on clinical symptoms, full blood count, urinalysis, depending on the suspected focus) and enrolment will take place when antibiotics are initiated. We will enroll 50 participants on each of the oral antimicrobials (amoxicillin, azithromycin, ciprofloxacin). Patients being initiated on antimicrobial agents will be randomly selected, screened, and enrolled. Prescription of antibiotics will be determined by the managing clinician according to standard clinic guidelines. Participants will be requested to take the antibiotic therapy at the same time daily. At baseline, data will be collected on age, sex, gastrointestinal disorders (e.g., diarrhea and vomiting), weight, height, alcohol use, illicit drug use, and details of concomitant medications, including antiretroviral regimens. A blood sample will be drawn for measurement of serum creatinine at baseline. Table 1 demonstrates all study procedures.

**Table 1.** Study procedures.

Protocol Activity	Screen and enrolment	Day 2 +/-3days	End antibiotic Treatment	Week 2	Week 4	Week 6	Week 8
Informed Consent	x						
Clinical evaluation	x	x	x	x	x	x	X
**Laboratory**							
Serum creatinine	x						
ALT, total Bilirubin****				x **			
PK blood draw*		x		x **	x **		
Sputum cultures		x		x **	x **	x **	x **

** for participants on TB treatment where applicable

***Participant follow-up***

Study visits will be conducted at enrolment, on day 2 for measurement of drug concentrations and the last day of antibiotic use to determine treatment outcome (after 5 – 7 days).

***Sparse pharmacokinetic sampling for antimicrobial drugs***

Sampling for measurement of antibiotic concentrations will be performed on day 2 of antimicrobial therapy. Directly observed therapy will be performed on the day of the blood draw. For those on oral azithromycin and ciprofloxacin, a pre-dose sample (trough concentrations) will be drawn, while for those on amoxicillin, a pre-dose and 6-hour blood draw will be taken. ( Table 2)

**Table 2.** TDM for antimicrobials.

Drug	Administration	Time of blood draw
Amoxycillin (+/- clavulanic acid)	Oral	Pre-dose, 6 hours post dose
Azithromycin	Oral	Pre-dose (trough),
Ciprofloxacin	Oral	Pre-dose (trough),

***Intense pharmacokinetic sampling for antimicrobial drugs***

Intensive pharmacokinetic sampling will be conducted after at least 2 days of oral antibiotic use, for 20 randomly selected participants on each of these oral drugs; amoxicillin, ciprofloxacin, and azithromycin. At least 20 participants were chosen based on the target of having less than 20% relative standard error in the estimated coefficient of variation. For amoxicillin, blood will be drawn pre-dose, 0.5 h, 1 h, 1.5 h, 2h, 2.5 h, 3 h, 4h, 6h, and 8 h post-dose. For azithromycin and ciprofloxacin, blood will be pre-dose, and at 1 h, 2 h, 3h, 4h, 5h, 6h, 8h, 12h, and 24h post-dose dosing.

### Patients on intravenous antibiotics (Ceftriaxone)

Patients who are acutely ill and admitted in the in-patient wards of Mulago hospital will be enrolled for pharmacokinetic sampling while on ceftriaxone. Blood draws will occur when a patient has received at least 1 dose. A blood draw will be taken prior to dosing (Ctrough) and again, 2 hours after completion of bolus administration for ceftriaxone. Participants will be followed up to determine treatment outcome at the end of treatment or at discharge.

### Participants on anti-tuberculosis treatment

The current tuberculosis (TB) treatment involves fixed dose combinations based on weight bands, including an intensive phase of rifampicin, isoniazid, ethambutol, and pyrazinamide (RHEZ) for 2 months, followed by a continuation phase of isoniazid and rifampicin (HR) for 4 months. Dosing is determined by weight bands, with 3, 4, or 5 tablets of RHZE or HR depending on weight (<55, 55–69, ≥70 respectively). Each tablet contains 150 mg of rifampicin and 75 mg of isoniazid for both combinations. Fixed dose combinations in the intensive phase include concomitant pyrazinamide and ethambutol.

**Figure 1.**
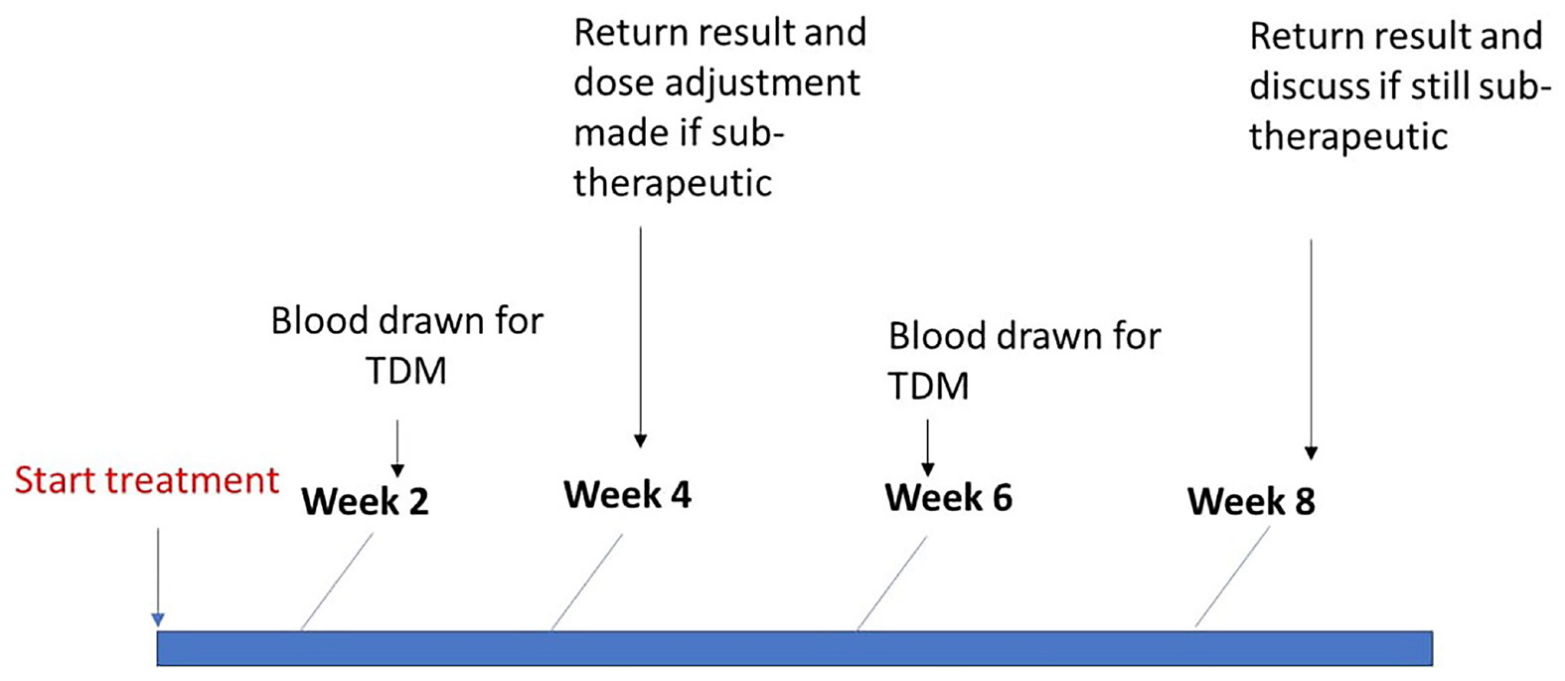
Therapeutic drug monitoring procedure with dose adjustment for participants on TB drugs.

Serum rifampicin and isoniazid concentrations (C2hr, C4hr, C6hr) will be measured in participants when fasting, and following directly observed therapy, after two weeks of treatment (First TDM). Results of blood concentrations will be received within 24–48 hours and assessed to determine if the C _max_ and AUC is within the therapeutic targets. Sub-therapeutic C _max_ concentrations are considered less than 8mg/L and 3mg/L for rifampicin and isoniazid, respectively. AUCs for rifampicin and Isoniazid will also be considered and compared with existing published therapeutic ranges ^22^. Participants found to have subtherapeutic drug exposures will have dose escalation and be asked to take one additional pill of the drug that did not achieve therapeutic target concentrations (rifampicin, isoniazid or both) ( Table 3). A second blood draw for measurement of drug concentrations will be performed after another two weeks (Second TDM) and dose adjustment performed again based on the guidance in Table 3, if concentrations are still below the target. ( Figure 1)

**Table 3.** Dose adjustment table for isoniazid and rifampicin in patients with HIV.

Weight band	Normal drug levels	Sub-target INH <3mg/dl Normal RIF	Normal INH Sub-target RIF <8mg/dl	Sub-target INH <3mg/dl and Sub-target RIF <8mg/dl
<55kg	Continue INH 225 mg and RIF 450 mg (3 FDCs)	Give an additional INH tablet equivalent to: INH 300 mg	Give an additional rifampicin tablet equivalent to 600 mg dose	Give additional rifampicin and isoniazid tablets or FDCs equivalent to 300 mg INH and 600 mg rifampicin
55–69kg	Continue INH 300 mg and RIF 600 mg (4 FDCs)	Give an additional INH tablet equivalent to INH 375 mg dose	Give an equivalent rifampicin tablet equivalent to 750 mg dose	Give additional rifampicin and isoniazid tablets or FDCs equivalent to 375 mg INH and 750 mg rifampicin
≥70kg	Continue INH 375 mg and RIF 750 mg (5 FDCs)	Give an additional INH tablet equivalent to 450 mg dose	Give an additional rifampicin tablet equivalent to 900 mg dose	Give additional rifampicin and isoniazid tablets or FDCs equivalent to 450 mg INH and 900 mg rifampicin

If the participant is still not achieving the therapeutic target concentrations after the second C _max_, another 1hr and 6hr blood draw will be conducted to assess for the presence of fast or delayed absorption. An additional dose adjustment with 1 additional tablet of the respective drug may be prescribed for these patients at the discretion of the study team and with consideration of observed side effects, treatment response, and concentrations attained. Those who attain therapeutic drug concentrations during TDM will revert to the previous dose. The final doses provided will be recorded for all participants.

***Sputum analysis***

For participants with tuberculosis, sputum cultures will be conducted at the Makerere University Microbacteriology laboratory. Cultures using Mycobacterium Growth Indicator tube (MGIT) and Lowenstein-Jensen (LJ) agar will be conducted within 7 days of enrolment and will be conducted every two weeks for the first eight weeks of TB treatment (intensive phase).

### Laboratory measurement of drug concentrations and sample handling

After blood sample collection, blood samples will immediately be placed in a dark cooler box and transported to the laboratory within 30 minutes of collection, after which samples will be centrifuged, aliquoted, batched, and frozen at -80ªC until quantification of drug concentrations is done. Quantification of the drug concentrations will be performed using pre-established validated liquid chromatography mass spectrometry (LC-MS) methods and high-performance liquid chromatography with ultraviolet detection (HPLC-UV).

### Safety assessment

A creatinine level will be measured in patients who undergo intensive pharmacokinetic blood draws, acutely ill in-patients and out-patients without a recent measurement. All other safety testing will be performed according to standard of care. Participants who are found to be pregnant during follow-up will not be excluded, however, pharmacokinetic data will be censored at that point.

Adverse event (AE) assessment and reporting will be conducted for participants undergoing dose adjustments for tuberculosis treatment. All observed or volunteered AEs, regardless of suspected causal relationship, will be reported. Adequate information will be obtained to evaluate the causality and severity of the AE using the Naranjo score and Division of AIDS (DAIDS) table respectively. Following adverse events, follow-up will be conducted until the event or its sequelae resolve or stabilize. Participants who suffer harm will be managed by the study which has provided trial insurance for those receiving dose adjustment.

### Statistical analysis

***Analysis of data on antimicrobial drugs: sparse pharmacokinetic data***

Sparse pharmacokinetic data for antimicrobials will be analysed as medians with standard deviations. We assess for the effect of covariates like age, sex, body-mass index, CD4 cell counts on drug concentrations

***Analysis of data on antimicrobial drugs: intensive pharmacokinetic data***

Non-linear mixed-effects models will be used to calculate the pharmacokinetic parameters of the drugs and this will include the maximum plasma concentration (Cmax), time to maximum plasma concentration (Tmax), and area under the plasma concentration-time curve (AUC). We will also explore the effects of covariates like age, sex, concomitant medications, and body-mass index on the pharmacokinetic parameters.

***Pharmacodynamic parameters***

We will use pharmacokinetic and pharmacodynamic models to assess the effect of the pharmacokinetic parameters on clinical response. For sparse pharmacokinetic data, we will use drug concentrations as a covariate in the linear regression models predicting clinical response. Clinical response will be defined as either clinical cure (resolution of symptoms) or clinical failure (lack of improvement in signs and symptoms of infection or recurrence of signs/symptoms of infection after initial improvement or death considered at least possibly due to the infection.

***Analysis of data on anti-tuberculosis drugs***

For participants on anti-tuberculosis drugs, we will evaluate whether dose adjustment leads to the achievement of therapeutic targets.

As an exploratory objective, using Kaplan Meier survival analysis, time to sputum culture negativity will be compared for those who received dose adjustment and those who did not receive dose adjustment within this study. We will also utilize the SOUTH dataset where drug concentrations of rifampicin and isoniazid were measured (without dose adjustment), where 78% and 84% of participants had rifampicin and isoniazid concentrations below the therapeutic targets respectively.

### Data Monitoring Committee

A Data Monitoring Board (DMB) will be established to evaluate data for participants undergoing TDM with dose adjustment. The board, consisting of an independent chair and members with expertise in clinical trials, will be responsible for safeguarding participant safety and ensuring study integrity. Periodic data reviews will be conducted by the DMB to monitor toxicities, and any concerns identified will be communicated to the study team. The following events, among others, may prompt a request to analyze available safety data for participants receiving dose adjustment and to generate a recommendation on termination of TDM; 1) three participants receiving dose adjustment experience a grade 4 or 5 adverse event in the same system organ class or considered similar (excluding non-clinically significant laboratory abnormalities) that is assessed as probably related or as related to the experimental treatment and 2) one subject receiving dose adjustment experiences a Grade 5 adverse event that is assessed as related to the experimental treatment.

## Dissemination of results

These results will be presented to both local and international stakeholders through meetings and international conferences. The raw dataset can be made available to other researchers upon request and signing data sharing agreements, after final results have been published.

## Confidentiality

Clinical data will be entered into the study specific dataset by designated staff on a regular basis. Case Record Forms and other source documents will be kept in locked cabinets. No participant identifying information will be disclosed in any publication or at any conference activities arising from the study.

## Discussion

Obtaining optimal drug concentrations is vital in treatment success whereas sub-optimal drug concentrations can lead to consequences such as AMR, treatment failure, and adverse outcomes.

This study will increase our understanding of the concentrations of commonly used antibiotics including amoxicillin, azithromycin, ciprofloxacin, ceftriaxone in PLWH, who are at high risk of recurrent infections such as urinary and respiratory tract infections. Understanding the pharmacokinetics and pharmacodynamics of these drugs will increase our understanding of the rising burden of AMR in PLWH, and possibly pave way for interventions that directly impact drug administration.

Therapeutic drug monitoring is increasingly being used for antibiotic dose optimization in the attempt to improve the attainment of pharmacokinetic and pharmacodynamics targets and outcomes of severe infections including in critically ill patients. Although there is a great need of TDM for monitoring selected antibacterial use, there is limited data on its applicability in ensuring appropriate pharmacokinetic and pharmacodynamics outcomes in resource-limited settings. Data from this study will contribute to informing clinicians and researchers about the practicability of using TDM in real-life settings for monitoring the achievement of target concentrations, and the utility of dose adjustment following TDM in different sub-groups of patients such as those with TB and patients with HIV on ART.

This study has some limitations. We cannot ascertain adherence to all treatment provided since directly observed therapy is not conducted throughout treatment, however, we will conduct directly observed therapy on the days when blood samples are drawn for measurement of drug concentrations. In addition, there are several factors that affect pharmacokinetics that we may not be able to assess during this study, for example pharmacokinetics during pregnancy, drug interactions with unknown medications provided over the counter, and comorbidities that occur less frequently and may lead to physiological changes.

In conclusion, the findings from this study will provide information on drug concentrations of frequently used antimicrobial agents in PLWH and guide clinical practices for optimizing doses of rifampicin and isoniazid in order to work towards appropriate dosing and improved treatment outcomes among PLWH.

## Ethics and consent

This study has been approved by the Infectious Diseases Institute Research and Ethics Committee (IDI REC) (IDI-REC-2023-83) on 26th February, 2024 and approvals for these changes will be sought prior to implementation. Written informed consent will be obtained by the study team before any study-related activities are conducted. The study will adhere to the Declaration of Helsink. This publication adhered to the SPIRIT guidelines for reporting study protocols.

## Data Availability

No data are associated with this article. The extended data associated with this study are available in the Open Science Framework (OSF) titled “Therapeutic drug monitoring for antimicrobial agents for people living with HIV (TAP), https://doi.org/10.17605/OSF.IO/3QE4M ^11^. This project contains the following extended data: Participant information sheet and consent form. SPIRIT checklist. Data are available under the terms of the Creative Commons Zero "No rights reserved" data waiver (CC0 1.0 Public domain dedication).
