# Supplementary material for: Therapeutic drug monitoring for antimicrobial agents for people living with HIV (TAP)
Source: Wellcome Open Res. 2025 Apr 22;9:694. Originally published 2024 Nov 26. [Version 2] doi: 10.12688/wellcomeopenres.22707.2 (PMC12107230; doi:10.12688/wellcomeopenres.22707.2)
Supplement: Peer review history

# Reviewer report: Reviewer response for version 2

**DOI:** 10.21956/wellcomeopenres.26588.r122582

**Published:** 2025-05-26

**Authors:** Jessica Cusato

**Affiliations:** 1 University of Turin, Turin, Italy

The study aims to generate data to support personalized antimicrobial and anti-TB dosing in PLWH.

 In fact, it is a prospective study addressing antimicrobial resistance in PLWH, focusing on antibiotic exposure and drug-drug interactions, particularly during TB treatment. The authors measured plasma concentrations of selected antibiotics (e.g., amoxicillin, rifampicin, isoniazid) using validated chromatographic methods and apply TDM with dose adjustments in a TB-treated subgroup.

**To the Authors**

 1. The article deals with the TDM of antimycobacterials in PWH with tuberculosis. I suggest revising the title accordingly (it should be clearly stated the study will focus only on antimicrobial agents used for the treatment of TB).

 2. Table 2 is not acceptable in the present form. Please add information on the time of blood draw for all the 6 antibiotics that will be subjected to TDM

 3. Table 3 provides dose adjustment advice for isoniazid and rifampicin based on TDM results for PWH with lower drug exposure. What about dose adjustments in patients with rifampicin or isoniazid concentrations above the upper therapeutic threshold (i.e. rifampicin > 24 mg/L)?

Is the study design appropriate for the research question?

Yes

Is the rationale for, and objectives of, the study clearly described?

Yes

Are sufficient details of the methods provided to allow replication by others?

Yes

Are the datasets clearly presented in a useable and accessible format?

Partly

Reviewer Expertise:

Pharmacology

I confirm that I have read this submission and believe that I have an appropriate level of expertise to confirm that it is of an acceptable scientific standard.

# Reviewer report: Reviewer response for version 1

**DOI:** 10.21956/wellcomeopenres.25009.r116607

**Published:** 2025-02-05

**Authors:** Lambert Assoumou

**Affiliations:** 1 INSERM, Pierre Louis Institute of Epidemiology and Public Health,, Sorbonne University, Paris, France

The main aims of this study are to determine the concentrations of selected antimicrobial agents (amoxicillin, azithromycin, ciprofloxacin and ceftriaxone) in people living with HIV (PLWH) who require antimicrobial therapy, and to assess the usefulness of therapeutic drug monitoring (TDM) in achieving therapeutic goals in PLWH receiving rifampicin and isoniazid for the treatment of tuberculosis. Two primary endpoints are planned: a pharmacokinetic endpoint and a pharmacodynamic endpoint (resolution of signs/symptoms). The study will also explore the effects of covariates such as age, gender, concomitant medications and body mass index on pharmacokinetic parameters.

 The study will recruit adult PLWH, aged 18 or over, with suspected or confirmed bacterial infection receiving at least one of the following antibiotics: amoxicillin, azithromycin, ciprofloxacin, rifampicin, isoniazid or ceftriaxone. For participants on anti-tuberculosis treatment, a dose adjustment will be made for those who have not reached target therapeutic concentrations of rifampicin, isoniazid or both at any time during the follow-up period based on TDM.

 50 participants on each of the oral antimicrobials (amoxicillin, azithromycin, ciprofloxacin) and 25 participants for intravenous antibiotics (ceftriaxone) and anti-TB drugs (rifampicin and isoniazid) were expected.

**Here is my comments :**

 The study has two primary endpoints, however, the sample size calculation was based solely on the pharmacokinetic endpoint. With such a sample size for each antibiotic, what would be the power to achieve the pharmacodynamic endpoint (sign/symptom resolution)?

 The method used to diagnose bacterial infections and tuberculosis must be indicated.

 The authors should explain why patients who discontinue antibiotics during the study should be excluded from the analysis. In my opinion, only participants who discontinue antibiotics without follow-up assessment should be excluded from the analysis. In addition, for women who become pregnant during the course of the study, pharmacokinetic data should be censored at the date of pregnancy, but pharmacodynamic data should be evaluated throughout the duration of the study.

Is the study design appropriate for the research question?

Yes

Is the rationale for, and objectives of, the study clearly described?

Yes

Are sufficient details of the methods provided to allow replication by others?

Partly

Are the datasets clearly presented in a useable and accessible format?

Not applicable

Reviewer Expertise:

NA

I confirm that I have read this submission and believe that I have an appropriate level of expertise to confirm that it is of an acceptable scientific standard, however I have significant reservations, as outlined above.

# Response

**Published:** 2025-04-09

**Authors:** Isabella Kyohairwe

**Affiliations:** Research, Makerere University Infectious Diseases Institute, Kampala, Central Region, Uganda

**Comment:** The method used to diagnose bacterial infections and tuberculosis must be indicated. **Response:** TB is diagnosed using GeneXpert and CXR however only those who are GeneXpert positive are enrolled. For bacterial infections, those with suspected or confirmed bacterial infection, based on the managing clinicians decision, were enrolled when antibiotics were started. Bacterial infection is largely  diagnosed using clinical symptoms and a full blood count, urine dip stick, depending on the suspected focus – line 125 

**Comment**: The authors should explain why patients who discontinue antibiotics during the study should be excluded from the analysis. In my opinion, only participants who discontinue antibiotics without follow-up assessment should be excluded from the analysis. In addition, for women who become pregnant during the course of the study, pharmacokinetic data should be censored at the date of pregnancy, but pharmacodynamic data should be evaluated throughout the duration of the study. **Response:** Yes, data from the participants who get pregnant will be analysed and only the PK data will be censored from the point of pregnancy. Line 211 The decision to exclude those who discontinued antibiotics was made so as to have complete PK data for the majority of the patients and avoid having data with no matching PK parameters. We cannot change the exclusion of those whose antibiotics were discontinued at this point since the study is almost completed. However, we have not had to exclude anyone for this reason up to this point.

# Reviewer report: Reviewer response for version 1

**DOI:** 10.21956/wellcomeopenres.25009.r115966

**Published:** 2025-01-13

**Authors:** Rubeshan Perumal

**Affiliations:** 1 Centre for the AIDS Programme of Research in South Africa, KwaZulu Natal, South Africa

The investigators propose an important and timely interrogation of the pharmacokinetics and dose-optimisation of commonly used antimicrobial and anti-tuberculous agents in people living with HIV receiving ART. Overall, the proposal is well-written, describes the project in sufficient detail, and advances the important scientific and public health goals of mitigating antimicrobial resistance and optimising the treatment of infections(including TB) in people with HIV. The following comments may help to strengthen the manuscript/protocol:

**Abstract**

 The objective, as stated in the Abstract, needs to be rewritten for greater clarity. At present, it reads as though the concentrations of antimicrobial drugs will be assessed in people living with HIV and TB (receiving ART and rifampicin-based TB treatment). However, the study aims to evaluate the pharmacokinetics of commonly used antimicrobials (amoxicillin, azithromycin, ciprofloxacin, ceftriaxone), rifampicin, and isoniazid) in people with HIV receiving ART.

 “This study was reviewed and approved by the research and ethics committee in February 2024. Enrolment is projected to begin by August 2024.” Has the trial already started recruiting? Perhaps this needs to be updated to reflect the present status.

**Introduction**

 The introduction is well-written and provides a clear rationale for pursuing the proposed work on therapeutic drug monitoring for people with HIV on TB treatment. However, a stronger rationale for evaluating the PK and drug-drug interactions of the commonly prescribed antimicrobial drugs is necessary. Suggestions include:

1. The scale of prescription of common antimicrobial agents in people living with HIV in Uganda and sub-Saharan Africa.
2. The known population PK profiles of these commonly prescribed antimicrobial agents. This should include outcomes for the most common indications (e.g. community-acquired pneumonia) in this population and any evidence to suggest that suboptimal drug concentrations may be contributory to the suboptimal outcomes. Please also include whether PK/exposure targets exist for these drugs.
3. Since the central theme of this project is the prevention/mitigation of antimicrobial resistance, it would be helpful for the introduction to include some empirical data on the drivers of resistance to the specific antimicrobial agents of interest in this study. To what extent is the problem known to be driven by PK/PD mismatch compared to other mechanisms of AMR?
4. The known and potential drug-drug interactions between common antimicrobials and ART should be presented.

**Protocol and methods**

 The overall study design and methods reflect high levels of scientific rigour and appropriateness.

1. Study objective 2 should include “impact on clinical outcomes” – this could be sputum culture conversion or WHO-defined end-of-treatment outcomes.
2. A brief justification for using trough concentrations as the PK parameter endpoint for each antimicrobial drug is necessary.
3. Using clinical responses to the commonly used antimicrobial agents is pragmatic but may be heavily confounded by the indications for the treatment and the appropriateness of the prescription. The authors should consider whether focusing their attention and effort on the most common indication/s for these drugs would be more useful. 
4. While the study makes provision for people receiving intravenous medication, it may be best to limit this study to ambulatory patients receiving oral treatment for all drugs except ceftriaxone.
5. Studying the pharmacokinetics, drug-drug interactions, and PK/PD in the context of an acute diarrheal illness may be very challenging. If this is an exclusion criterion, it should be included in the exclusion criteria. If it is not an exclusion criterion, a brief section on how this will be handled in interpreting the data is necessary.
6. The sample size section should include a final statement on the proposed sample size. The total sample size appears to be 200 participants (50 for amoxicillin, 50 for ciprofloxacin, 50 for azithromycin, 25 for ceftriaxone, and 25 for TB treatment).
7. Co-treatment with more than one of these agents will likely occur (e.g. ceftriaxone plus azithromycin, or amoxicillin-clavulanate plus azithromycin). Would this be permissible?
8. At present, the antiretroviral therapy regimen is not specified. Allowing *any *ART regimen is not practical and will limit the ability to study drug-drug interactions, in particular. Would it be possible to restrict this study to one (or at most, two) ART regimen/s to improve the methodological rigour?
9. Have the investigators considered a control group of people who are HIV-negative and, therefore, not receiving ART? Without this group, it will be challenging to determine how any antimicrobial PK data relates to co-administration with ART (i.e., drug-drug interaction).

**Therapeutic drug monitoring with dose-adjustment**

 The proposed study of therapeutic drug monitoring for optimising the treatment of tuberculosis is an attractive application of TDM for clinical impact and the mitigation of AMR.

1. In people with HIV, rifampicin Cmax has been shown to occur later than expected (C4hr or C6hr) in a significant proportion of people. It would be helpful if a justification for the C2hr timepoint is provided for this particular setting.
  1. What proportion of people with HIV from this setting has been shown to have Tmax at 2 hours vs 4 hours vs 6 hours?
  2. What is the risk of increasing the dose based on a low C2hr in a patient with Cmax at 4 or 6 hours, for example? It may be worthwhile to mention some of the rapidly accumulating data on the safety of higher doses of rifampicin. The use of a DMC and stopping rules, as proposed, does strengthen this study,

Once again, the investigators are congratulated for proposing this highly relevant and timely study. The TDM component, in particular, responds to a significant gap in the present therapeutic approach to TB.

Is the study design appropriate for the research question?

Yes

Is the rationale for, and objectives of, the study clearly described?

Yes

Are sufficient details of the methods provided to allow replication by others?

Yes

Are the datasets clearly presented in a useable and accessible format?

Not applicable

Reviewer Expertise:

Clinical Pharmacology; TB/HIV Epidemiology; Drug-resistant tuberculosis; TB/HIV Treatment Research

I confirm that I have read this submission and believe that I have an appropriate level of expertise to confirm that it is of an acceptable scientific standard.

# Response

**Published:** 2025-04-09

**Authors:** Isabella Kyohairwe

**Affiliations:** Research, Makerere University Infectious Diseases Institute, Kampala, Central Region, Uganda

We are grateful to the reviewers whose comments will improve the description of this study.

**Reviewer:** The objective, as stated in the Abstract, needs to be rewritten for greater clarity. At present, it reads as though the concentrations of antimicrobial drugs will be assessed in people living with HIV and TB (receiving ART and rifampicin-based TB treatment). However, the study aims to evaluate the pharmacokinetics of commonly used antimicrobials (amoxicillin, azithromycin, ciprofloxacin, ceftriaxone), rifampicin, and isoniazid) in people with HIV receiving ART.

**Response: **In the abstract, the objective of this study is to determine the concentrations of selected antimicrobial agents in people living with HIV requiring antimicrobial therapy and to assess the utility of therapeutic drug monitoring in achieving therapeutic targets for PLWH receiving rifampicin and isoniazid for the treatment of tuberculosis. This implies that we shall evaluate the PK of antibiotics in participants living with HIV. Those with TB will enrolled in the TDM arm.

**Comment:** “This study was reviewed and approved by the research and ethics committee in February 2024. Enrolment is projected to begin by August 2024.” Has the trial already started recruiting? Perhaps this needs to be updated to reflect the present status.

**Response: **This is true. We have adjusted the statement to indicate that enrollment began in September 2024.

**Reviewer** **:** The scale of prescription of common antimicrobial agents in people living with HIV in Uganda and sub-Saharan Africa.

**Response: **Antibiotics are commonly prescribed for prophylaxis and management of other conditions among people living with HIV. The rates of prescription may vary across different countries in the region and range from 40% in Nigeria to as high as 85% in Tanzania (1). In Uganda, this data remain limited but antibiotic prescription is highly prevalent as shown by a recent survey conducted across 13 hospitals in the country (2).  This has been added on line 25-28 in the revised manuscript.

**Reviewer:** The known population PK profiles of these commonly prescribed antimicrobial agents. This should include outcomes for the most common indications (e.g. community-acquired pneumonia) in this population and any evidence to suggest that suboptimal drug concentrations may be contributory to the suboptimal outcomes. Please also include whether PK/exposure targets exist for these drugs.

**Response: **Thank you for this important comment. The PK profile of these antibiotics in our study population in Uganda has not previously been described, and we have added a section about known PK targets for the antibiotics covered in this study starting on line 41. According to the Ugandan Ministry of Health data, at least 25 children die of pneumonia every day. Among adults, pneumonia remains a major cause of morbidity and mortality, particularly in people living with HIV. For example, in Uganda, studies at Mulago Hospital have shown that a microbial cause was identified in over 90% of HIV-positive adults with pneumonia, with tuberculosis and pneumococcus being the most common pathogens. Outcome data for urinary tract infections, which are also a common indication for antibiotics, is also limited. However, at Mulago National Referral Hospital, 30% of people who received urine cultures were resistant to ampicillin (line 34). It is not yet established whether this could have been attributed to sub-optimal exposures. While specific PK/exposure targets exist for some antibiotics (e.g., trimethoprim-sulfamethoxazole), for many agents commonly used in our setting, such as ampicillin and ceftriaxone, there is limited local data on whether these targets are consistently achieved in people living with HIV. This is an important area for future research, especially given the potential for altered drug metabolism in this population.

**Reviewer:** Since the central theme of this project is the prevention/mitigation of antimicrobial resistance, it would be helpful for the introduction to include some empirical data on the drivers of resistance to the specific antimicrobial agents of interest in this study. To what extent is the problem known to be driven by PK/PD mismatch compared to other mechanisms of AMR?

**Response** **We thank the reviewer for the comment. Brusse-Keizer et al. demonstrated that suboptimal exposure to amoxicillin was associated with poor outcomes in patients with COPD** **(3)** **. Resistance to fluoroquinolones has also been linked to subinhibitory drug concentrations** **(4)** **,** **as has treatment outcomes of anti-TB drugs** **(5)** **. That said, it is also true that other mechanisms like genetic mutations and horizontal gene transfer also play a significant role in antimicrobial resistance. We have expanded the introduction to reflect these points, starting from line 37 in the revised manuscript.** **Reviewer:** The known and potential drug-drug interactions between common antimicrobials and ART should be presented.

**Response:** While ceftriaxone and penicillins like amoxicillin may not have significant interactions with ART, certain ARVs cause enzyme inhibition with resultant increase in antibiotic drug levels and risk of toxicity. For example, Lopinavir/ritonavir is predicted to cause inhibition of P-gp and MRP2 and could potentially increase azithromycin exposure with risk of QT interval prolongation and torsades des points (6). No clinical PK studies have been done, and no dose adjustments are currently recommended. We have added some more to this background on lines 40 to 45 in the revised manuscript.

**Protocol and methods**

 The overall study design and methods reflect high levels of scientific rigour and appropriateness.

**Reviewer:** Study objective 2 should include “impact on clinical outcomes” – this could be sputum culture conversion or WHO-defined end-of-treatment outcomes.

**Response:** This study is aimed at describing PK data for antibiotics such as amoxicillin. We acknowledge that it is not powered to assess PD outcomes in individuals receiving these antibiotics. However, exploratory PK-PD data will be available for those on tuberculosis treatment, as they will have biweekly cultures from which colony counts can be utilized.

**Reviewer:** A brief justification for using trough concentrations as the PK parameter endpoint for each antimicrobial drug is necessary.

**Response:** Using through concentrations will help determine if the organisms are consistently exposed to concentrations above historical MIC. In addition to the sparsely sampled participants, we will intensively sample a subgroup of participants. By combining these data using population pharmacokinetics (PopPK) modeling techniques, we will be able to estimate the overall drug exposure for all participants, including those who were sparsely sampled. This approach leverages the benefits of PopPK analysis while reducing the need for extensive sampling and resource-intensive drug concentration assays. Combining both datasets will provide a more comprehensive understanding of the PK of these drugs in HIV patients within our setting.

**Reviewer:** Using clinical responses to the commonly used antimicrobial agents is pragmatic but may be heavily confounded by the indications for the treatment and the appropriateness of the prescription. The authors should consider whether focusing their attention and effort on the most common indication/s for these drugs would be more useful. 

**Response:** We agree with the reviewer, however the aim of this study is to describe the PK of these drugs in HIV and evaluate if we can utilize these models in routine practice when antibiotics are given. However, there is another parallel study taking place as part of this consortium where appropriateness and indications of the antibiotic use will be described and can inform these results. ( https://doi.org/10.12688/wellcomeopenres.23532.1)

**Reviewer:** While the study makes provision for people receiving intravenous medication, it may be best to limit this study to ambulatory patients receiving oral treatment for all drugs except ceftriaxone.

**Response: **We thank the reviewer for this comment, however at this point in time, we have completed enrollment of patients receiving ceftriaxone. Since this is the most frequently prescribed intravenous antibiotic in Uganda, we believe the PK data will still be informative.

**Reviewer** **:** Studying the pharmacokinetics, drug-drug interactions, and PK/PD in the context of an acute diarrheal illness may be very challenging. If this is an exclusion criterion, it should be included in the exclusion criteria. If it is not an exclusion criterion, a brief section on how this will be handled in interpreting the data is necessary.

**Response: **Knowledge of acute diarrheal disease is important and we will review it as a covariate if this was present while interpreting the PK data especially for those who have unexpectedly low concentrations.

**Reviewer** **:** The sample size section should include a final statement on the proposed sample size. The total sample size appears to be 200 participants (50 for amoxicillin, 50 for ciprofloxacin, 50 for azithromycin, 25 for ceftriaxone, and 25 for TB treatment).

**Response:** This is true, the total sample size is 200 and this has been updated on line 155.

**Reviewer** **:** Co-treatment with more than one of these agents will likely occur (e.g. ceftriaxone plus azithromycin, or amoxicillin-clavulanate plus azithromycin). Would this be permissible? **Response:** This is very likely and we are enrolling those on multiple drugs of interest and recording this data so that it can be interpreted accordingly.

**Reviewer** **:** At present, the antiretroviral therapy regimen is not specified. Allowing *any *ART regimen is not practical and will limit the ability to study drug-drug interactions, in particular. Would it be possible to restrict this study to one (or at most, two) ART regimen/s to improve the methodological rigour?

**Response:** We agree with the reviewer. Most patients in Uganda are on dolutegravir based regimens with a few receiving efavirenz. This is given mostly with tenofovir and lamivudine. We will therefore have enrolled participants on all these ART and will analyze the data with this as a potential covariate.

**Reviewer** **:** Have the investigators considered a control group of people who are HIV-negative and, therefore, not receiving ART? Without this group, it will be challenging to determine how any antimicrobial PK data relates to co-administration with ART (i.e., drug-drug interaction). **Response**: This is very useful feedback, and we believe having HIV-negative patients would have been very informative. Although this is not possible at this point, we plan to compare the antibiotic exposures in our study with previously published data (including those not on ART).

**Therapeutic drug monitoring with dose-adjustment**

 The proposed study of therapeutic drug monitoring for optimising the treatment of tuberculosis is an attractive application of TDM for clinical impact and the mitigation of AMR.

**Reviewer:** In people with HIV, rifampicin Cmax has been shown to occur later than expected (C4hr or C6hr) in a significant proportion of people. It would be helpful if a justification for the C2hr timepoint is provided for this particular setting. What proportion of people with HIV from this setting has been shown to have Tmax at 2 hours vs 4 hours vs 6 hours? What is the risk of increasing the dose based on a low C2hr in a patient with Cmax at 4 or 6 hours, for example? It may be worthwhile to mention some of the rapidly accumulating data on the safety of higher doses of rifampicin. The use of a DMC and stopping rules, as proposed, does strengthen this study,

**Response: **The reviewer is correct. We acknowledge that some patients may not have peak concentrations at C2hr due to delayed absorption. Therefore, increasing rifampicin doses in this population may lead to adverse drug reactions. The protocol was amended such that blood draws will also be taken at C4hr and C6hr for all patients on TB treatment as previously published (7). We will therefore be able to estimate the Cmax and overall exposure (are under the curve) more accurately to determine who requires dose adjustment.
